# Cancer History and Subjective Sleepiness in Obstructive Sleep Apnea: A Real-World Observational Study

**DOI:** 10.3390/jcm15124573

**Published:** 2026-06-12

**Authors:** Giulia Sartori, Claudia Di Chiara, Andrea Gretter, Alberto Fantin, Ernesto Crisafulli

**Affiliations:** 1 Respiratory Medicine Unit, Department of Medicine, University of Verona and Azienda Ospedaliera Universitaria Integrata of Verona, Largo L.A. Scuro, 10, 37134 Verona, Italy; giulia.sartori.verona@gmail.com (G.S.); claudiadichiara95@gmail.com (C.D.C.); gretterandrea@gmail.com (A.G.); af@albertofantin.com (A.F.); 2 Department of Pulmonology, S. Maria della Misericordia University Hospital, 33100 Udine, Italy

**Keywords:** obstructive sleep apnea, daytime sleepiness, Epworth Sleepiness Scale, cancer history, symptom perception, cancer survivors

## Abstract

**Background/Objectives:** Daytime sleepiness in obstructive sleep apnea (OSA) shows substantial variability and is not fully explained by disease severity. This study aimed to evaluate whether a history of cancer is associated with subjective daytime sleepiness independently of respiratory burden. **Methods**: In this observational cohort study, 402 untreated patients with OSA were included. Cancer history was defined as a documented diagnosis of malignancy prior to baseline polygraphy. Daytime sleepiness was assessed using the Epworth Sleepiness Scale (ESS). Multivariable linear and logistic regression models were used to evaluate the association between cancer history and ESS (continuous and dichotomized as >10), adjusting for age, sex, body mass index, smoking status, chronic obstructive pulmonary disease, and apnea–hypopnea index (AHI). Sensitivity analyses additionally adjusted for heart disease and nocturnal hypoxic burden. **Results:** Sixty-two patients (15%) had a history of cancer. OSA severity and hypoxemia indices were comparable between groups. In multivariable analysis, cancer history was independently associated with modestly lower ESS scores (B = −1.66, 95% confidence interval [CI] −2.96 to −0.37; *p* = 0.012) and a reduced likelihood of excessive daytime sleepiness (ESS > 10) (odds ratio [OR] = 0.25, 95% CI 0.07 to 0.85; *p* = 0.027). **Conclusions:** In this real-world cohort of untreated patients with OSA, cancer history is associated with modestly lower subjective daytime sleepiness despite comparable disease severity, supporting a potential dissociation between physiological burden and symptom perception. These findings indicate that reliance on subjective sleepiness alone may contribute to under-recognition of clinically relevant OSA in patients with a cancer history.

## 1. Introduction

Obstructive sleep apnea (OSA) is a highly prevalent sleep-related breathing disorder characterized by recurrent upper airway obstruction during sleep, leading to intermittent hypoxia and sleep fragmentation [1]. Daytime sleepiness is a common and clinically relevant consequence of OSA, although its underlying mechanisms are heterogeneous and include sleep deprivation, sleep fragmentation, and other sleep disorders [2]. Patients with moderate-to-severe OSA are more likely to experience excessive daytime sleepiness, and the severity of daytime sleepiness has been associated with an increased risk of cardiovascular outcomes [3]. Despite this, substantial variability in daytime sleepiness exists among patients with similar OSA severity, suggesting that factors beyond respiratory indices contribute to symptom perception [4,5]. The Epworth Sleepiness Scale (ESS) is widely used to assess subjective sleepiness and has demonstrated good reliability in clinical and research settings [6]. However, ESS captures sleep propensity rather than fatigue and may be influenced by individual perception and contextual factors [7].

Increasing attention has been given to the relationship between OSA and cancer [8]. Intermittent hypoxia and sleep fragmentation have been proposed as mechanisms linking OSA to tumor progression and mortality through pathways involving oxidative stress, inflammation, and immune dysregulation [9,10]. Conversely, patients with a history of cancer frequently report sleep disturbances, fatigue, and altered symptom perception, which may persist long after treatment completion [11,12]. Cancer-related fatigue and psychological factors may influence how symptoms are experienced and reported, potentially leading to a dissociation between objective physiological impairment and subjective symptom burden [13,14]. Notably, fatigue and sleepiness are distinct but overlapping constructs, and their differentiation may be particularly challenging in cancer survivors [15]. This raises the possibility that standard tools such as the ESS may not fully capture symptom burden in this population.

Whether a history of cancer modifies the perception of daytime sleepiness in patients with OSA, independently of disease severity, remains unclear. Therefore, the aim of the present study was to evaluate the association between cancer history and subjective daytime sleepiness in a cohort of patients with OSA, while accounting for objective measures of disease severity and relevant clinical confounders.

## 2. Methods

### 2.1. Study Cohort

Between September 2020 and July 2025, we retrospectively analyzed prospectively collected data of subjects referred for suspected sleep-disordered breathing to the dedicated outpatient clinic of the Respiratory Medicine Unit at the Azienda Ospedaliera Universitaria Integrata of Verona.

Exclusion criteria were (1) age <18 years; (2) absence of OSA; (3) ongoing ventilatory treatment, including continuous positive airway pressure (CPAP); (4) polygraphy performed for indications other than suspected sleep-disordered breathing (e.g., neuromuscular disease); (5) missing or poor-quality respiratory polygraphy data; and (6) cancer diagnosis occurring after the sleep study.

Cancer history was defined as a documented diagnosis of malignancy prior to the sleep study, including diagnoses made in the same calendar year.

Information regarding cancer stage, disease activity (active disease versus remission), ongoing oncological treatments, and prior exposure to chemotherapy, radiotherapy, immunotherapy, or hormonal therapy was not systematically available in the clinical database and therefore could not be included in the analyses.

The study protocol was approved by the local ethics committee and conducted in accordance with the Declaration of Helsinki and Good Clinical Practice guidelines.

### 2.2. General Measures

Demographic and anthropometric characteristics included age, sex, body mass index (BMI), and neck circumference. Comorbidities included arterial hypertension, heart disease, diabetes, and chronic obstructive pulmonary disease (COPD). Smoking status was also recorded.

### 2.3. Nocturnal Polygraphy and Daytime Sleepiness

Respiratory polygraphy was performed using a portable Nox T3s^TM^ device (Nox Medical, Reykjavík, Iceland; https://noxmedical.com). Only recordings with adequate signal quality were included. Recorded signals included peripheral oxygen saturation (SpO_2_), thoracic and abdominal movements (inductive belts), nasal airflow derived from the belts, snoring, and body position. Signals were analyzed using the Noxturnal^TM^ software version 6.1 (Nox Medical, Reykjavík, Iceland) with an automated respiratory scoring algorithm and subsequently reviewed by an experienced pulmonologist specialized in sleep medicine. Respiratory events were scored according to the American Academy of Sleep Medicine criteria [16]. Apnea was defined as a ≥90% reduction in airflow for ≥10 s and hypopnea as a ≥30% reduction in airflow associated with ≥3% oxygen desaturation. The apnea–hypopnea index (AHI) was calculated as the number of apneas and hypopneas per hour of recording (events/h). AHI categories were classified as mild (AHI 5–15 events/h), moderate (15–30 events/h), and severe (>30 events/h) [1,16].

Pulse oximetry parameters included oxygen desaturation index (ODI; number of ≥3% desaturations per hour), lowest SpO_2_ (nadir), mean desaturation depth, and time spent with SpO_2_ < 90% (T_90_).

Daytime sleepiness was assessed using the Italian version of the ESS [17], an eight-item self-reported questionnaire evaluating the likelihood of falling asleep in daily situations. Scores range from 0 to 24, with ESS > 10 indicating excessive daytime sleepiness.

### 2.4. Statistical Analysis

Continuous variables are presented as median (interquartile range [IQR]) and categorical variables as number and percentage. Comparisons between patients with and without cancer history were performed using the Mann–Whitney U test for continuous variables and the chi-square or Fisher’s exact test for categorical variables, as appropriate. Standardized mean differences (SMDs) were calculated to assess baseline imbalance, with values ≥0.20 indicating meaningful imbalance.

The primary outcome was ESS as a continuous variable. The association between cancer history and ESS was assessed using multivariable linear regression analysis, including age, sex, BMI, smoking status, COPD, and AHI as covariates. Covariates were selected a priori on the basis of clinical plausibility and their known association with both cancer history and sleepiness perception [5,18,19,20]. As a secondary analysis, excessive daytime sleepiness (ESS > 10) was evaluated using multivariable logistic regression with the same covariates. Linear regression results are reported as unstandardized coefficients (B) with standard errors (SEs) and 95% confidence intervals (CIs), while logistic regression results are reported as odds ratios (ORs) with 95% CIs. Model calibration for logistic regression was assessed using the Hosmer–Lemeshow goodness-of-fit test.

Sensitivity analyses were performed by additionally adjusting for heart disease and, separately, for nocturnal hypoxic burden (T_90_). Additional sensitivity analyses were performed by including adjustment for cardiovascular and metabolic comorbidities (arterial hypertension, heart disease, and diabetes) and using alternative ESS cut-offs (>8, >9, >11, and >12) to evaluate the robustness of the findings. To explore potential effect modification, interaction terms between cancer history and age, as well as AHI, were included in the models. Among patients with cancer, the association between time from cancer diagnosis to sleep study and ESS was explored using Spearman correlation. Multicollinearity was assessed using variance inflation factors. Analyses involving ESS were conducted as complete-case analyses.

A two-sided *p*-value < 0.05 was considered statistically significant. All analyses were performed using IBM SPSS Statistics, version 30 (IBM Corp., Armonk, NY, USA).

## 3. Results

**Figure 1:**
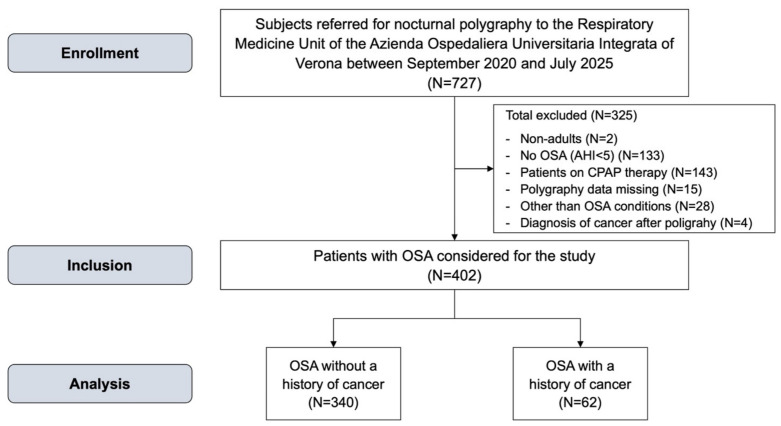
Study flowchart. *Abbreviations:* OSA indicates obstructive sleep apnea; CPAP, continuous positive airway pressure.

The study flow diagram is presented in Figure 1.

### 3.1. Study Population and Baseline Characteristics

A total of 402 patients with OSA were included in the analysis, of whom 62 (15%) had a history of cancer. Baseline characteristics are reported in Table 1.

Patients with cancer were significantly older than those without cancer (median 71 vs. 61 years; *p* < 0.001; SMD = 0.90). Cardiovascular and metabolic comorbidities, including arterial hypertension, heart disease, and diabetes, were more prevalent among patients with cancer (all *p* < 0.05). In contrast, sex distribution, BMI, smoking status, and COPD prevalence were comparable between groups.

Importantly, indices of OSA severity and nocturnal hypoxemia were well balanced between groups. No significant differences were observed in AHI, ODI, nadir SpO_2_, mean desaturation, or T_90_, with all corresponding SMD values < 0.10, indicating a comparable physiological burden of OSA.

### 3.2. Daytime Sleepiness According to Cancer History

**Figure 2:**
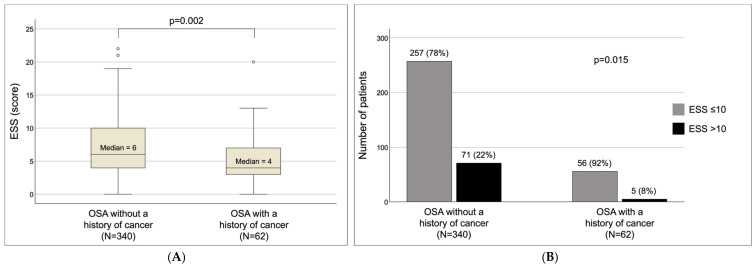
Daytime sleepiness according to cancer history in patients with OSA: (**A**) Distribution of Epworth Sleepiness Scale (ESS) scores in patients with and without a cancer history. Median values are indicated within each box. (**B**) Prevalence of excessive daytime sleepiness (ESS > 10) in the two groups. *p*-values were calculated using the Mann–Whitney U test (**A**) and the chi-square test (**B**). *Abbreviations:* ESS, Epworth Sleepiness Scale; OSA, obstructive sleep apnea.

The distribution of ESS scores according to cancer history is shown in Figure 2A. Patients with a history of cancer had significantly lower ESS values (median 4 [IQR 4]) compared with those without cancer (median 6 [IQR 6]) (*p* = 0.002).

The prevalence of excessive daytime sleepiness was also significantly lower among patients with cancer (8%) compared with those without cancer (22%) (*p* = 0.015), as shown in Figure 2B.

### 3.3. Association Between Cancer History and Daytime Sleepiness

In multivariable linear regression analysis (Table 2), cancer history was independently associated with lower ESS scores (B = −1.66, 95% CI −2.96 to −0.37; *p* = 0.012), after adjustment for age, sex, BMI, smoking status, COPD, and AHI.

Among covariates, age and AHI were significantly associated with ESS, whereas sex, BMI, smoking status, and COPD were not.

No significant interactions were observed between cancer history and age (*p* = 0.417) or AHI (*p* = 0.922), indicating that the association between cancer history and ESS was consistent across age groups and OSA severity levels.

Among patients with cancer, ESS was not associated with time since cancer diagnosis (Spearman’s ρ = 0.036, *p* = 0.785).

### 3.4. Excessive Daytime Sleepiness

Excessive daytime sleepiness was observed in 67 patients. In multivariable logistic regression analysis (Table 3), cancer history was associated with a significantly lower likelihood of excessive daytime sleepiness (OR = 0.25, 95% CI 0.07 to 0.85; *p* = 0.027).

AHI was independently associated with excessive daytime sleepiness, whereas age, sex, BMI, smoking status, and COPD were not.

### 3.5. Cancer Characteristics

Cancer characteristics are summarized in Table 4. The most common malignancies were genitourinary and hematologic tumors. Cancer site categories were not mutually exclusive, and 21% of patients had a history of more than one malignancy. The median time from cancer diagnosis to polygraphy was 8 years (IQR 14).

### 3.6. Sensitivity Analyses

Sensitivity analyses confirmed the robustness of the main findings. After additional adjustment for heart disease, cancer history remained independently associated with lower ESS scores in both linear (B = −1.65, *p* = 0.013) and logistic models (OR = 0.25, *p* = 0.027) (Table 5).

Similarly, adjustment for nocturnal hypoxic burden (T_90_) did not materially change the results, and cancer history remained associated with lower ESS scores (B = −1.61, *p* = 0.015) (Table 6).

Further adjustment for cardiovascular and metabolic comorbidities did not materially change the results, and cancer history remained independently associated with lower ESS scores (B = −1.71, *p* = 0.010).

The strongest association was observed at higher ESS thresholds (e.g., ESS > 11), while estimates at lower thresholds were attenuated (Table 7).

## 4. Discussion

In this real-world cohort study, a history of cancer was independently associated with modestly lower subjective daytime sleepiness in patients with OSA, despite comparable objective measures of disease severity. Notably, indices of respiratory disturbance and nocturnal hypoxemia, including AHI, ODI, and T_90_, were similar between groups, indicating that the physiological burden of OSA did not differ according to cancer history. These findings suggest a dissociation between objective disease severity and subjective symptom perception in this population.

The variability of daytime sleepiness among patients with OSA is well recognized and only partially explained by respiratory indices [2,5]. Notably, subjective sleepiness may be uncoupled from objective disease severity in specific clinical populations [3,21]. Accordingly, patients with similar AHI values may report markedly different levels of sleepiness, reflecting the contribution of biological, psychological, and behavioral factors to symptom perception [4,5,21]. Our findings extend this concept by identifying cancer history as a potential modifying factor.

The interaction between OSA and cancer has been increasingly investigated, particularly regarding the potential role of intermittent hypoxia in tumor biology. Experimental and clinical evidence suggests that intermittent hypoxia may promote tumor progression through oxidative stress, inflammation, and immune dysregulation [9]. However, most studies have focused on the impact of OSA on cancer outcomes [8,9,10,11], whereas the potential influence of cancer on the clinical expression of OSA has been less explored.

In our cohort, patients with cancer were older and had a higher prevalence of cardiovascular and metabolic comorbidities, consistent with the epidemiology of cancer survivors [22]. Importantly, the association between cancer history and lower ESS scores persisted after adjustment for general characteristics and OSA severity and remained robust across multiple sensitivity analyses, including adjustments for cardiovascular comorbidities and hypoxic burden. The direction of the association was consistent across multiple ESS cut-offs, although statistical significance was not observed at all thresholds, likely reflecting reduced power at more extreme cut-off points. In general, older age has been associated in some studies with lower reporting of subjective daytime sleepiness despite a substantial burden of sleep-disordered breathing [23]. Although age was included in all multivariable models, and no significant interaction between age and cancer history was observed, we cannot exclude that age-related differences in symptom perception may have contributed to the observed findings. Moreover, no interaction was observed with OSA severity, and ESS was not associated with time since cancer diagnosis, suggesting that the observed effect is not limited to a specific phase of the oncological trajectory but may represent a more stable alteration in symptom perception. Together, these findings support the robustness of the observed association and suggest that it is not explained by confounding factors or limited to specific patient subgroups. Importantly, our results do not indicate a protective effect of cancer on OSA severity but rather a difference in symptom perception [12,13,15].

Several mechanisms may explain these findings. First, cancer-related fatigue is a multidimensional construct that differs from sleepiness and may overlap with it, potentially leading to underreporting of sleep propensity when assessed using instruments such as the ESS [13]. Cancer survivors frequently report persistent fatigue, neurocognitive symptoms, and psychological distress, which may alter the perception and reporting of symptoms [15,24,25]. In this context, the ESS may underestimate sleepiness in patients with a history of cancer. Additionally, a “response shift” phenomenon—where patients adjust their internal standards and expectations following a major illness—may contribute to reduced reporting of symptoms [26]. These findings support the concept that subjective sleepiness may not reliably reflect disease burden in specific clinical populations [20].

An important consideration is the distinction between fatigue and sleepiness [24]. Cancer survivors frequently experience chronic cancer-related fatigue, which is characterized by physical and mental exhaustion but does not necessarily increase sleep propensity [24]. Consequently, patients may report substantial fatigue while maintaining relatively low ESS scores. Because fatigue-specific instruments were not available in the present study, we cannot determine whether lower ESS values reflected genuinely lower sleepiness or a different symptom-reporting pattern in which fatigue predominated over sleep propensity. Therefore, the present findings should be considered hypothesis-generating and require confirmation using objective measures of sleepiness as well as validated assessments of cancer-related fatigue.

Biological mechanisms may also play a role. Cancer and its treatments are associated with chronic inflammation and alterations in cytokine signaling, including interleukin (IL)-1, IL-6, and tumor necrosis factor (TNF)-α, which may affect central sleep-wake regulation [27]. Persistent low-grade inflammation and increased sympathetic activity have also been described in cancer survivors and may contribute to altered neurophysiological responses and symptom perception [28,29].

An alternative explanation relates to healthcare pathways and referral patterns. Patients with a history of cancer may be more closely monitored and referred for sleep evaluation at lower symptom thresholds, potentially leading to the identification of OSA in individuals with a lower subjective symptom burden [30]. This may contribute to the observed dissociation between physiological severity and reported sleepiness.

From a clinical perspective, these findings have important implications. Daytime sleepiness is often used as a key indicator for OSA diagnosis and treatment decisions [1,21]. If patients with a history of cancer report lower levels of sleepiness despite comparable disease severity, reliance on subjective symptoms alone may lead to under-recognition of clinically relevant OSA in routine clinical practice. Clinicians should therefore consider a more comprehensive assessment of OSA risk in patients with a cancer history, even in the absence of prominent sleepiness. Together, these findings highlight the need for a more comprehensive assessment of OSA beyond subjective sleepiness in specific clinical populations [21] and may be particularly relevant in routine clinical practice, where reliance on subjective symptoms often guides referral and diagnosis.

This study has important strengths, including a well-characterized clinical cohort, a comprehensive assessment of OSA severity, and consistent findings across multiple analytical approaches. The use of standardized mean differences further supports the comparability of physiological indices between groups. An additional strength of this study is the inclusion of untreated patients, minimizing the potential confounding effects of CPAP therapy or wake-promoting medications on subjective sleepiness.

This study has several limitations. First, its observational design precludes causal inference. Second, detailed information regarding cancer stage, remission status, active disease, and exposure to specific oncological treatments (including chemotherapy, radiotherapy, immunotherapy, and hormonal therapies such as androgen deprivation therapy) was not systematically available. These factors may substantially influence fatigue, sleep quality, inflammation, and symptom perception and therefore could have affected ESS scores. Third, daytime sleepiness was assessed using a subjective questionnaire, which may be influenced by psychological and behavioral factors and may underestimate symptoms in cancer populations. Although widely used, the ESS is subject to substantial intra-individual variability. Previous studies have shown that repeated ESS measurements over short intervals may vary by >5 points in approximately 15–23% of individuals [31,32,33] and by >8 points in a smaller proportion of cases [34]. Therefore, the relatively small difference observed in our study (2 points in median ESS) may fall within the expected variability of the instrument, and its clinical relevance should be interpreted with caution. Fourth, ESS was assessed at a single time point, and repeated measurements were not available, which may have introduced measurement error. Fifth, information on habitual sleep duration was not available. Sleep duration has been associated with both daytime sleepiness and cancer risk, and its absence may represent a source of residual confounding. Finally, the number of patients with excessive daytime sleepiness in the cancer group was relatively small, which may limit the precision of logistic regression estimates, and the number of head and neck cancers was very limited, precluding site-specific analyses [35].

## 5. Conclusions

In patients with untreated OSA, a history of cancer is associated with modestly lower self-reported ESS scores despite comparable disease severity. Although the magnitude of the difference is small, these findings suggest a dissociation between physiological burden and symptom perception and highlight that reliance on subjective sleepiness alone may lead to under-recognition of clinically relevant OSA in patients with a history of cancer. However, our findings should be interpreted as hypothesis-generating and may reflect differences in symptom perception or reporting rather than true differences in physiological sleepiness.

## Figures and Tables

**Table 1:** General and nocturnal polygraphy data characteristics of patients with OSA according to cancer history.

Variables	Total Cohort (N = 402)	OSA Without a History of Cancer (N = 340)	OSA with a History of Cancer (N = 62)	SMD	*p*-Value
Age, years	63 [19]	61 [19]	71 [15]	0.90	**<0.001**
Male, n (%)	315 (78)	264 (78)	51 (82)	0.12	0.417
BMI, kg/m^2^	31.5 [9.6]	31.8 [9.3]	30.1 [8.9]	0.20	0.216
Neck circumference, cm	42 [5]	42 [5]	41 [6]	0.07	0.512
Former or current/never-smokers, n (%)	207(62)/127(38)	171(61)/110(39)	36(68)/17(32)	0.16	0.331
Arterial hypertension, n (%)	258 (64)	210 (62)	48 (77)	0.34	**0.018**
Heart disease, n (%)	137 (34)	106 (31)	31 (50)	0.39	**0.004**
Diabetes, n (%)	100 (25)	77 (23)	23 (37)	0.32	**0.015**
COPD, n (%)	69 (17)	56 (16)	13 (21)	0.12	0.388
AHI, events/h	18.6 [24.6]	18.2 [24.3]	21.2 [24.3]	0.01	0.327
AHI categories, n (%)					0.158
Mild	172 (43)	150 (44)	22 (36)	0.18	
Moderate	109 (27)	94 (28)	15 (24)	0.08	
Severe	121 (30)	96 (28)	25 (40)	0.26	
ODI, events/h	22.8 [29.3]	21.8 [30]	28.1 [24.8]	0.01	0.343
Nadir SpO_2_, %	78 [12]	78 [13]	79 [12.3]	0.09	0.628
Mean desaturation, %	5.3 [2.8]	5.3 [2.9]	5.1 [2.5]	0.06	0.736
T_90_, %	10.7 [30]	9.6 [30.4]	14.1 [24.9]	0.06	0.743

The data are reported as the number of patients (percentage) or medians [interquartile range]. Percentages are calculated on available data. Significant values are shown in bold. Standardized mean differences (SMDs) were calculated using means and standard deviations (SDs) to quantify baseline imbalance between groups; values ≥0.20 were considered indicative of meaningful imbalance. *Abbreviations:* OSA indicates obstructive sleep apnea; BMI, body mass index; COPD, chronic obstructive pulmonary disease; AHI, apnea–hypopnea index; ODI, oxygen desaturation index; SpO_2_, pulse oximetry oxygen saturation; T_90_, percentage of time spent with oxygen saturation below 90%.

**Table 2:** Multivariate linear regression analysis.

Independent Variables	B (Unstandardized Coefficient)	SE	95% CI for B	t	*p*
Cancer history	−1.66	0.66	−2.96 to −0.37	−2.52	**0.012**
Age	−0.044	0.020	−0.083 to −0.004	−2.17	**0.031**
Sex	−0.92	0.58	−2.07 to 0.22	−1.59	0.113
BMI	0.018	0.035	−0.050 to 0.086	0.524	0.601
Smoking status	−0.41	0.51	−1.41 to 0.59	−0.80	0.424
COPD	0.39	0.65	−0.88 to 1.68	0.61	0.545
AHI	0.038	0.013	0.013 to 0.063	2.96	**0.003**

Bold text indicates a statistically significant difference. *Abbreviations:* SE, standard error; CI, confidence interval; BMI, body mass index; COPD, chronic obstructive pulmonary disease; AHI, apnea–hypopnea index.

**Table 3:** Multivariate logistic regression model (dependent variable: ESS > 10).

Variables	OR	95% CI	*p*-Value
Cancer history, yes	0.25	0.07 to 0.85	**0.027**
Age	0.98	0.96 to 1.01	0.363
Sex	0.73	0.34 to 1.55	0.408
BMI	0.99	0.96 to 1.04	0.980
Smoking status, current/former	0.59	0.32 to 1.09	0.096
COPD, yes	1.09	0.47 to 2.52	0.846
AHI	1.02	1.01 to 1.03	**0.013**

Reference categories: no cancer; never-smokers; no COPD. Hosmer–Lemeshow test *p* = 0.249. In bold are reported significant values. *Abbreviations:* OR, odds ratio; CI, confidence interval; BMI, body mass index; COPD, chronic obstructive pulmonary disease; AHI, apnea–hypopnea index.

**Table 4:** Cancer characteristics.

Cancer Sites (History), Not Mutually Exclusive	
Lung, n (%)	6 (9.7)
Breast, n (%)	5 (8.1)
Colorectal (bowel), n (%)	7 (11.3)
Genitourinary (kidney + bladder + prostate), n (%)	26 (41.9)
Endocrine (thyroid + pituitary), n (%)	6 (9.7)
Hematologic, n (%)	15 (24.2)
Other solid tumors, n (%)	8 (12.9)
Patients with ≥2 malignancy, n (%)	13 (21)
Median time from cancer diagnosis to polygraphy, years [IQR]	8 [14]

*Abbreviation:* IQR, interquartile range. Cancer history was abstracted from clinical records; site categories reflect documented malignancy sites and may include multiple primary cancers in the same patient.

**Table 5:** Sensitivity analyses (multivariate linear (**A**) and logistic (**B**)) for the presence of heart disease.

**A**					
**Independent Variables**	**B** **(Unstandardized Coefficient)**	**SE**	**95% CI for B**	**t**	***p***
Cancer history	−1.65	0.66	−2.95 to −0.35	−2.51	**0.013**
Age	−0.035	0.021	−0.077 to −0.007	−1.65	0.099
Sex	−1.04	0.59	−2.19 to 0.12	−1.76	0.079
BMI	0.024	0.035	−0.044 to 0.092	0.69	0.491
Smoking status	−0.37	0.51	−1.38 to 0.63	−0.74	0.462
COPD	0.47	0.65	−0.82 to 1.76	0.72	0.473
AHI	0.037	0.013	0.012 to 0.063	2.91	**0.004**
Heart disease	−0.65	0.52	−1.67 to 0.36	−1.27	0.207
**B**					
**Variables**		**OR**	**95% CI**	***p*** **-Value**	
Cancer history, yes		0.25	0.07 to 0.85	**0.027**	
Age		0.99	0.97 to 1.02	0.548	
Sex		0.69	0.32 to 1.49	0.345	
BMI		1.001	0.96 to 1.04	0.951	
Smoking status, current or former		0.60	0.33 to 1.11	0.103	
COPD, yes		1.11	0.48 to 2.59	0.804	
AHI		1.02	1.01 to 1.03	**0.014**	
Heart disease, yes		0.77	0.40 to 1.50	0.445	

Reference categories: no cancer; never-smokers; no COPD; no heart disease. Hosmer–Lemeshow test *p* = 0.178. In bold are reported significant values. *Abbreviations:* SE, standard error; CI, confidence interval; BMI, body mass index; COPD, chronic obstructive pulmonary disease; AHI, apnea–hypopnea index; OR, odds ratio.

**Table 6:** Sensitivity analyses (multivariate linear) for the hypoxic burden (T_90_).

Independent Variables	B (Unstandardized Coefficient)	SE	95% CI for B	t	*p*
Cancer history	−1.61	0.66	−2.91 to −0.31	−2.45	**0.015**
Age	−0.047	0.020	−0.086 to −0.007	−2.31	**0.022**
Sex	−0.91	0.58	−2.05 to 0.24	−1.56	0.119
BMI	0.003	0.036	−0.069 to 0.074	0.071	0.943
Smoking status	−0.41	0.51	−1.41 to 0.59	−0.80	0.425
COPD	0.24	0.66	−1.06 to 1.54	0.36	0.720
AHI	0.033	0.013	0.007 to 0.059	2.47	**0.014**
T_90_	0.014	0.010	−0.007 to 0.035	1.34	0.181

Bold text indicates a statistically significant difference. *Abbreviations:* T_90_, percentage of time spent with oxygen saturation below 90%; SE, standard error; CI, confidence interval; BMI, body mass index; COPD, chronic obstructive pulmonary disease; AHI, apnea–hypopnea index.

**Table 7:** Sensitivity logistic regression models using alternative ESS cut-offs.

Dependent variable: ESS > 8			
Variables	OR	95% CI	*p*-value
Cancer history, yes	0.58	0.26 to 1.30	0.190
Age	0.98	0.96 to 1.01	0.121
Sex	0.54	0.27 to 1.08	0.082
BMI	1.01	0.97 to 1.04	0.660
Smoking status, current/former	0.82	0.47 to 1.41	0.466
COPD, yes	1.59	0.79 to 3.81	0.190
AHI	1.01	1.00 to 1.01	**0.045**
Hosmer–Lemeshow test *p* = 0.209			
Dependent variable: ESS > 9			
Variables	OR	95% CI	*p*-value
Cancer history, yes	0.45	0.18 to 1.51	0.096
Age	0.98	0.96 to 1.01	0.081
Sex	0.57	0.28 to 1.18	0.132
BMI	0.99	0.95 to 1.03	0.584
Smoking status, current/former	0.66	0.37 to 1.17	0.159
COPD, yes	1.35	0.63 to 2.90	0.439
AHI	1.02	1.01 to 1.03	**0.020**
Hosmer–Lemeshow test *p* = 0.798			
Dependent variable: ESS > 11			
Variables	OR	95% CI	*p*-value
Cancer history, yes	0.22	0.05 to 0.95	**0.043**
Age	0.99	0.97 to 1.02	0.581
Sex	0.70	0.30 to 1.64	0.412
BMI	1.01	0.97 to 1.06	0.624
Smoking status, current/former	0.71	0.36 to 1.40	0.318
COPD, yes	1.32	0.54 to 3.25	0.543
AHI	1.02	1.01 to 1.04	**0.005**
Hosmer–Lemeshow test *p* = 0.065			
Dependent variable: ESS > 12			
Variables	OR	95% CI	*p*-value
Cancer history, yes	0.33	0.07 to 1.46	0.144
Age	0.99	0.96 to 1.02	0.472
Sex	0.51	0.17 to 1.44	0.200
BMI	1.02	0.97 to 1.07	0.529
Smoking status, current/former	0.80	0.37 to 1.73	0.580
COPD, yes	1.33	0.49 to 3.65	0.578
AHI	1.02	1.01 to 1.04	**0.026**
Hosmer–Lemeshow test *p* = 0.463			

Reference categories: no cancer; never-smokers; no COPD. In bold are reported significant values. *Abbreviations:* ESS, Epworth Sleepiness Scale; OR, odds ratio; CI, confidence interval; BMI, body mass index; COPD, chronic obstructive pulmonary disease; AHI, apnea–hypopnea index.

## Data Availability

The datasets used and/or analyzed during the current study are available from the corresponding author upon reasonable request.
